# Bioactive Compounds of Strawberry and Blueberry and Their Potential Health Effects Based on Human Intervention Studies: A Brief Overview

**DOI:** 10.3390/nu11071510

**Published:** 2019-07-02

**Authors:** Katharina Miller, Walter Feucht, Markus Schmid

**Affiliations:** 1 Faculty of Life Sciences, Albstadt-Sigmaringen University, Anton-Günther-Str. 51, 72488 Sigmaringen, Germany; 2 Unit of Fruit Science, TUM School of Life Sciences Weihenstephan, Technical University of Munich, Dürnast 2, 85354 Freising, Germany

**Keywords:** anthocyanidins, polyphenols, berries, cardiovascular disease, inflammation

## Abstract

Strawberries and blueberries are two of the most commonly consumed berries. Berries, in general, are characterized by their highly nutritive compounds, including minerals, vitamins, fatty acids, and dietary fiber, as well as their high content and wide diversity of bioactive compounds, such as phenolic compounds and organic acids. These bioactive compounds have been associated with protective effects against chronic diseases, such as cardiovascular disease, cancer, Alzheimer’s and other disorders. In this paper 16 human intervention studies investigating the beneficial health effects of dietary strawberry or blueberry consumption on inflammation, cardiovascular disease or cognitive function and mental health are reviewed.

## 1. Introduction

Cardiovascular disease (CVD) is still the leading cause of death and disability in the world. CVDs describe numerous diseases involving the heart, brain, and blood vessels. Coronary heart disease (heart attack) and cerebrovascular disease (stroke) are two major CVDs caused by arteriosclerosis, the pathological process in the walls of blood vessels. In arteriosclerosis, deposits of fatty material and cholesterol (plaques) in blood vessels lead to narrow vessels and decreased blood flow. A blood clot can occur when the plaque ruptures. Depending on the place of development, in a coronary artery or in the brain, a blood clot can cause a heart attack or a stroke. The major metabolic risk factors for CVDs are overweight/obesity, raised blood pressure (hypertension), raised blood sugar (diabetes) and raised blood lipids (e.g., cholesterol). The occurrence and extent of these metabolic risk factors highly depend on behavioral risk factors like physical activity and diet. Overweight (BMI ≥ 25 kg/m^2^) and obesity (BMI ≥ 30 kg/m^2^) are the result of an imbalanced energy intake/usage and strongly related to the other metabolic risk factors. With increasing body mass index, the risk of coronary heart disease, ischaemic stroke, and type 2 diabetes mellitus increases [1].

A major contributing factor to the development, progression, and complication of chronic diseases, such as type 2 diabetes, CVD and Alzheimer’s, is a sustained pro-inflammatory state. Inflammation is known as the common, protective, and temporary response of the innate immune system to pathogens and injury stimuli, characterized by the production of pro- and anti-inflammatory cytokines. Cytokines are small soluble proteins, which act as signals between immune cells to coordinate the inflammatory response. Among cytokines, interleukin 1β (IL-1 β), interleukin-6 (IL 6) and tumor necrosis factor-alpha (TNF-α) are commonly induced together and act as pro-inflammatory and alarm cytokines [2,3,4]. In addition to the production of TNF-a, IL-1 and IL-6, endothelial dysfunction, oxidative stress in vascular endothelial cells, macrophage accumulation and formation of inflammasome characterize the inflammatory process, leading to atherogenesis, the plaque forming process [5]. Other biomarkers of inflammatory status, which are measured in humans, are acute phase proteins (i.e., C-reactive protein (CRP), fibrinogen, serum amyloid A), chemokines (i.e., monocyte chemoattractant protein (MCP)-1) and adhesion molecules (i.e., E-selectin, P-selectin, sVCAM-1). In addition to classic inflammatory stimuli, acute inflammatory stress can be induced by a single energy-dense meal providing a surplus of readily available carbohydrates and fat. In the long term, poor diet can lead to obesity, which is known as a chronic, low-grade inflammatory state [4]. A chronic, low-grade inflammatory state can lead to, for instance, tissue damage, endothelial dysfunction, thrombosis, insulin resistance, and high blood pressure, which can result in cardiovascular disease, diabetes, some forms of cancer, arthritis, neurodegenerative diseases, and many others [6].

Over 350 million people worldwide are affected by a major depressive disorder. Impaired cognitive and executive function are two common symptoms of depression. Executive function describes cognitive processes such as working memory, planning, problem-solving, cognitive flexibility, inhibitory control, directing attention, thoughts and, therefore, behaviors. Depressive symptoms, such as negative self-perception and low mood, are believed to be maintained by impaired executive function via perseveration and rumination. Consumption of flavonoids is associated with a decreased risk of developing depression [7].

Berries are characterized by their highly nutritive compounds, including minerals, vitamins, fatty acids, and dietary fiber. They also contain high contents and wide diversity of nonessential biologically active components, such as organic acids or polyphenols, with their subclasses, tannins and flavonoids [3,4,8]. Anthocyanins, a subclass of flavonoids, are important bioactive compounds in berries, which are responsible for the red-blue-purple coloring of berries [3,4]. Bioactive compounds are believed to have the ability to provide and activate cellular antioxidant protection, scavenge free radicals, inhibit inflammatory gene expression, and consequently protect against oxidant-induced and inflammatory cell damage and cytotoxicity. Therefore, bioactive compounds have been associated with protective effects against chronic diseases, such as cardiovascular disease (CVD), cancer, Alzheimer’s, depression, and other disorders [4,7,8,9].

Strawberries (*Fragaria*) and blueberries (*Vaccinium*) are two of the most demanded berries in fresh and frozen forms, as well as in processed and derived products like dried and canned forms, yogurts, beverages, jams, and jellies [3,10,11,12,13]. Numerous in vitro and in vivo studies demonstrated that amongst other foods strawberries and blueberries aid in providing and reducing the risk of developing several chronic diseases [2,13]. Strawberry and blueberry highly differ in the amount of different subclasses of bioactive compounds. The chemical compositions of strawberry and blueberry are compared in Table 1.

Literature research was carried out in June 2018. To review most current research, google scholar and the database Food Science and Technology Abstracts (FSTA) were used to identify topic related articles published later than January 2011. The searches used the following terms and text words alone and in combination: “strawberry”, “blueberry”, “berries”, “bioactive compounds”, “health effects”, “human intervention”, “inflammation”, “cardiovascular disease, “cognitive function” and “cancer”. In vitro studies, as well as in vivo studies conducted with animals, were excluded. In this paper 16 human intervention studies investigating the beneficial health effects of dietary strawberry or blueberry consumption are reviewed.

## 2. Strawberry

### 2.1. Composition

With about 60 mg per 100 g fresh fruit, strawberries are especially known for their high content of vitamin C. Strawberries also provide several other vitamins in lower extent, such as thiamine, riboflavin, niacin and vitamin B6 and contain fat-soluble vitamins, including carotenoids, vitamin A, vitamin E and vitamin K. Among fruits, strawberries are one of the richest natural folate sources (43 μg per 100 g fresh fruit) and show a remarkably high antioxidant activity. Strawberries are also a good source of manganese, iodine, magnesium, copper, iron, and phosphorus [3,15,16]. In addition, strawberries are rich in bioactive compounds, mainly represented by flavonoids, especially anthocyanidins, followed by phenolic acids like hydroxycinnamic, and hydroxybenzoic acids [14].

### 2.2. Inflammation

Seven clinical trials investigating the effects of strawberry intervention on inflammatory markers were identified. Among those seven studies, three tested postprandial effects and four long-term feeding effects on inflammation. Two studies were conducted with overweight participants, three with abdominally obese subjects, one with type two diabetic participants, and one with obese subjects with osteoarthritis.

In a cross-over design, the effect of strawberry antioxidants in beverage form on meal-induced postprandial inflammatory and insulin responses over a 6 h period has been investigated. Therefore, 24 overweight adults consumed a high-carbohydrate, moderate-fat meal, to increase markers of oxidative stress and insulin resistance, accompanied by either a single serving strawberry or a placebo beverage. The results showed a significant decrease in high sensitivity c-reactive protein (hs-CRP) and a reduction in postprandial insulin response (IL-6) by the strawberry beverage, but not by the placebo. No significant differences for plasminogen activator inhibitor (PAI)-1, IL-1 β and TNF- α concentrations were found among treatments [17]. In contrast, a similar study with the same 24 overweighed adults was conducted over six weeks and showed a significant attenuation of PAI-1 concentration and IL-1β response in the strawberry group, but no differences for platelet aggregation, hs-CRP, TNF-α, IL-6, insulin or glucose were found among treatments [18]. Despite a similar experimental set-up, Edirisinghe et al. [17] and Ellis et al. [18] reported different effects on postprandial PAI-1, IL-1β, IL-6, and hs-CRP concentrations by strawberry intake over different time periods. However, in both studies, an anti-inflammatory effect was found, though represented in a decrease of different biomarkers of inflammation.

On the other hand, two of the seven identified studies, conducted with abdominally obese subjects, observed no significant influence of strawberry consumption on tested inflammatory markers. In another 6-h postprandial study, 21 abdominally obese subjects with insulin resistance received a standard high-fat breakfast with one of four beverages containing 0, 10, 20 or 40 g freeze-dried strawberry (FDS). In this four-arm, placebo-controlled, 6-h postprandial, crossover study, Park et al. reported some effects on CVD related biomarker, as described in Section 2.3, but no influence on the measured inflammatory biomarker IL-6 was found [19]. In a seven-week double-blind, randomized, cross-over trial, 20 obese subjects received a prepared diet and either the strawberry powder (equivalent to four servings of 80 g frozen strawberry) or a placebo in form of a beverage. Zunino et al. reported no influence on cytokine concentrations of IL-1 β, IL-6, and TNF-α in the clinical trial [20]. The two studies of this section, showing no significant effects on inflammatory markers, primally investigated the effect of strawberry intervention on CVD and are therefore also listed in Section 2.3.

Basu et al. [21] (also listed in Section 2.3), Moazen et al. [22] and Shell et al. [23] reported anti-inflammatory effects of strawberry consumption in subjects with type II diabetes mellitus and obese subjects with osteoarthritis or elevated serum lipids. In a 12-week randomized, dose-response controlled trial, 60 subjects with abdominal adiposity and elevated serum lipids were assigned to consume one of four beverages: a low or high dose FDS beverage (25 g/day or 50 g/day) or a low or high dose calorie- and fiber-matched beverage. No effects on hs-CRP and adhesion molecules were observed among treatments. A significant decrease in serum malondialdehyde, a biomarker for oxidative stress, was found by both FDS-groups compared to control groups [21]. Thirty-six subjects with type II diabetes mellitus daily consumed either 50 g FDS beverage or a matched placebo beverage in a randomized, double-blind, controlled trial for six weeks. The results showed a significant increase of markers of total serum antioxidant status while serum malondialdehyde, glycated hemoglobin (Hb)A1c and hs-CRP concentrations significantly decreased. Serum glucose concentrations and anthropometric indices did not change significantly [22]. In double-blind crossover study 17 obese adults with osteoarthritis were randomly assigned to consume either an FDS beverage (50 g/day) or a control beverage daily for 12 weeks, respectively, with a two-week washout phase in between, to examine the effects of dietary strawberries on pain, markers of inflammation and quality of life indicators. The study showed a decrease in IL-6, IL-1, and matrix metalloproteinase, which are serum biomarkers of inflammation and cartilage degradation, after FDS beverage, but not after control beverage consumption. Moreover, constant, intermittent, and total pain were only significantly reduced by the FDS beverage consumption, but no differences for hs-CRP, nitrite, glucose, and lipid profiles were found [23].

Collectively, in five out of seven studies, an anti-inflammatory effect was presented by a decrease in some cytokine or acute phase protein concentrations or a decrease in oxidative stress. In two studies, no significant anti-inflammatory effect was observed.

### 2.3. Cardiovascular Disease

Several intervention studies provide a suggestion that berry fruit consumption has significant potential in the prevention and treatment of most risk factors mentioned above [6]. Coherent to the studies presented in Section 2.2, five human intervention studies investigating the effect of strawberry consumption on CVD-related markers were identified. Among those five studies, four were conducted with abdominal obese subjects and one with postmenopausal women. The major metabolic risk factors for CVDs are overweight/obesity, raised blood pressure (hypertension), raised blood sugar (diabetes), and raised blood lipids (e.g., cholesterol). Biomarkers for CVD, which are measured in humans, are, i.e., systolic and diastolic blood pressure, insulin, and glucose concentrations, distribution and size of blood lipids and antioxidant markers like plasma antioxidant capacity, whole blood glutathione, superoxide dismutase levels and serum catalase activity.

In addition to the results presented in Section 2.2, Park et al. reported a significant reduction in postprandial plasma insulin concentrations, insulin:glucose ratio, and rate of glucose and insulin increase in 21 abdominally obese subjects with insulin resistance, receiving a 40 g FDS beverage. A significant reduction in oxidized low-density lipoprotein was shown by the 20 g FDS beverage compared to all other beverages [19]. In addition to IL-1 β, IL-6, TNF-α concentrations, the seven-week double-blind, randomized, cross-over trial with 20 obese subjects, receiving a prepared diet and either the strawberry powder (equivalent to four servings of 80 g frozen strawberry) or a placebo observed no differences in blood pressure and medium and large HDL-cholesterol particles. However, a reduction in plasma concentrations of small HDL particles and small HDL-cholesterol, as well as an increase in LDL particle size was observed after the three-week strawberry intervention, but not the placebo intervention [20]. LDL and HDL particle distribution and size have been linked in previous studies to health status, in detail, to the metabolic syndrome and insulin resistance [20,24,25,26].

In a 12-week randomized, dose-response controlled trial, 60 subjects with abdominal adiposity and elevated serum lipids were assigned to consume one of four beverages: a low or high dose FDS beverage (25 g/day or 50 g/day) or a low or high dose calorie- and fiber-matched beverage. The results showed a significant reduction in serum total and LDL-cholesterol, and NMR-derived small LDL particles by the high FDS-dose compared to the low FDS-dose and the placebo beverages. No effects on any measures of adiposity, blood pressure, glycemia, and serum concentrations of HDL cholesterol and triglycerides were found [21]. In a similar study with the same experimental setup, Basu et al. reported a significant increase in plasma antioxidant capacity and whole blood glutathione at the end of the intervention by both FDS doses vs. controls. Other than the plasma antioxidant capacity, blood glutathione was significantly higher in the HD-FDS versus LD-FDS. Serum catalase activity was increased only in the LD-FDS compared to the controls. No effects on glutathione peroxidase and glutathione reductase enzyme activities, plasma copper, iron, selenium, and zinc, which also play an important role in antioxidant and pro-oxidant activities, were shown [27].

In an eight-week, randomized, double-blind, placebo-controlled, parallel intervention 60 postmenopausal women were either assigned to consume one of three beverages twice a day containing in total 0 g, 25 g or 50 g FDS. A decrease of systolic blood pressure and brachial- and femoral-ankle pulse wave velocity, a measured marker for arterial stiffness, was shown by 25 g FDS beverage compared to baseline but not to the other groups. An increase of plasma nitric oxide metabolite levels was shown by the 50 g FDS beverage but not the other beverages. In all three groups (0 g, 25 g or 50 g FDS) serum levels of superoxide dismutase increased at four weeks returning to baseline levels at eight weeks. No significant effect on diastolic blood pressure and metabolites were observed in all groups [28].

Collectively, the effect of strawberry consumption on cardiovascular disease has been examined in five intervention studies. Park et al. [19] and Basu et al. [27] reported anti-oxidant effects and Zunino et al. [20] and Basu et al. [21] reported some positive effects on distribution and size of blood lipids of strawberry consumption in obese subjects. In 60 postmenopausal women, a decrease in systolic blood pressure, arterial stiffness, and antioxidant effects were reported after eight-week strawberry intervention [28].

### 2.4. Cognitive Function and Mental Health

No studies investigating the effect of strawberry intervention on cognitive functions were identified during literature research.

## 3. Blueberry

### 3.1. Composition

Blueberries are nutritious fruits, as they are high in dietary fibers (3–3.5% of their fruit weight) and antioxidants, such as vitamin C, B complex, E, and A. Blueberries also provide high amounts of selenium, zinc, iron, and manganese and contain b-carotene, lutein, and zeaxanthin [9,12,13,29]. In addition, blueberries are rich in bioactive compounds, mainly represented by flavonoids, especially anthocyanidins and flavonols, as well as by phenolic acids like hydroxycinnamic acids [14].

### 3.2. Inflammation

Three clinical trials investigating the effects of blueberry intervention on markers, which are associated with inflammation, were identified. One study was conducted with subjects with metabolic syndrome, one with males with cardiovascular risk factors and one with women with at least two risk factors for type 2 diabetes.

In a double-blind, placebo-controlled study, 44 subjects with metabolic syndrome, consumed either a blueberry smoothie (yogurt and a milk-based smoothie with 22.5 g of freeze-dried blueberry powder added) or a placebo smoothie (identical smoothie without the blueberry powder) twice a day for six weeks. In the blueberry group, the endothelial function, expressed as reactive hyperemia index, was improved significantly more versus the placebo group [30]. On the other hand, no significant differences for markers of endothelial function and total plasma nitric oxide and soluble vascular adhesion molecule-were detected in a six-week randomized, placebo-controlled, crossover intervention with 18 male volunteers with cardiovascular risk factors receiving a wild blueberry drink (25 g freeze-dried powder, providing 375 mg of ACNs) or a placebo drink. However, in contrast to the placebo drink intake, the wild blueberry drink intake significantly reduced the levels of endogenously oxidized DNA bases, and the levels of H_2_O_2_-induced DNA damage in this study [31]. No influence on inflammatory markers, adhesion molecules, oxidative stress, and endothelial function were found in a single-blind, randomized, placebo-controlled, crossover design trial, 19 women with at least two risk factors for type 2 diabetes, who consumed 240 mL of wild blueberry juice or a placebo beverage for seven days [32].

Whereas Stull et al. [30] demonstrated an improvement in endothelial function after six-weeks blueberry consumption, Riso et al. [31] and Stote et al. [32] reported no significant influence on endothelial function and other inflammatory-related markers, with the exception of an antioxidant effect. The absence of an effect of blueberry intervention on inflammation studied by Stote et al. [32] could be based on the comparatively short intervention period of one week. Whereas Stull et al. and Riso et al. both conducted clinical trials over six weeks, the 44 subjects with metabolic syndrome received 45 g of freeze-dried blueberry powder per day (2 × 22.5 g) [30] and the 18 male subjects with cardiovascular risk factors received 25 g freeze-dried wild blueberry powder per day [31]. The different dose and type of blueberry powder intake, providing different amounts of bioactive compounds, could relate to the influence on endothelial function.

### 3.3. Cardiovascular Disease

In addition to markers of inflammation, the three clinical trials examined in Section 3.2 also investigated the influence of blueberry intervention on other CVD related markers like blood pressure, blood lipids, insulin sensitivity, etc. In addition to those three studies, a fourth study, conducted with postmenopausal women with pre- and stage 1- hypertension, has been identified for this section.

Besides the improvement of endothelial function in 44 subjects with metabolic syndrome consuming 22.5 g freeze-dried blueberry powder twice a day over six weeks, Stull et al. reported no difference in blood pressure and insulin sensitivity between the blueberry and placebo groups [30]. No influence on blood pressure (systolic and diastolic), as well as Framingham reactive hyperemia index and augmentation index were also found by Riso et al., where 18 male subjects with cardiovascular risk factors consumed 25 g wild blueberry freeze-dried powder for six weeks [31]. Stote et al. observed a trend for lowering systolic blood pressure and increased serum concentrations of nitrates and nitrites, an index of nitric oxide production, in 19 women with at least two risk factors for type 2 diabetes after one week 240 mL of wild blueberry juice. No significant changes in glucose, insulin, insulin sensitivity, triglycerides or blood pressure were detected [32]. In an eight-week, randomized, double-blind, placebo-controlled clinical trial, 48 postmenopausal women with pre- and stage 1- hypertension were randomly assigned to receive either 22 g freeze-dried blueberry powder or 22 g control powder in the form of a 480 mL drink. At the end of the intervention systolic blood pressure, diastolic blood pressure and brachial-ankle pulse wave velocity were significantly lower and Nitric oxide levels greater in the blueberry powder group compared with baseline values, whereas there were no changes in the group receiving the control powder [33].

In conclusion, these four intervention studies documented partially contrary findings on the effect of blueberry consumption on blood pressure, insulin sensitivity and oxidative stress. Three of four investigated clinical trials showed no significant effects of blueberry consumption on measured CVD related markers. Other than Stull et al. [30], Riso et al. [31] (six-week periods) and Stote et al. [32] (one-week period), Johnson et al. [33] reported a significant decrease in systolic and diastolic blood pressure after eight-week daily blueberry consumption.

### 3.4. Cognitive Function and Mental Health

Relating to epidemiological evidence, the consumption of flavonoids is associated with a decreased risk of developing depression, an attenuated rate of cognitive decline, and a reduced risk of dementia [7,34]. Three studies investigating the effects of blueberry intervention on cognitive function or mental health were identified. Two studies were conducted with healthy older adults and one with healthy young adults and children.

Two randomized, double-blind, placebo-controlled trials with twenty-one young adults (age: 20.1 ± 1.0 years) and fifty-two children (age: 8.2 ± 1.0 years) showed that the blueberry intervention (30 g freeze-dried wild blueberry with 30 mL low-flavonoid Rocks Orange Squash and 220 mL of water) increased post-consumption positive affect scores but had no effect on negative affect scores (PANS-NOW and PANS-C) compared to placebo intervention. There was also a significant main effect of session reduction of negative affect scores after both blueberry and placebo consumption [7]. Bowtell et al. investigated whether blueberry consumption improved brain perfusion, task-related activation, and cognitive function. Favorable effects on cerebrovascular and cognitive function in healthy older adults were shown in a randomized, double-blind, placebo-controlled trial after 12 weeks of blueberry concentrate supplementation (30 mL/day containing 387 mg anthocyanidins per day). Specifically, brain activation responses in several task related different areas and resting-state perfusion in the gray matter of the parietal and occipital lobes increased from baseline levels after 12 weeks of blueberry supplementation but not after placebo supplementation. A significant decrease in serum glutathione concentration was found in blueberry and placebo supplementation. No significant differences between the groups and no significant changes to baseline were found in brain activity, resting state perfusion in the frontal lobes, protein carbonylation HNE adduct or malonaldehyde formation and serum hs-CRP or BDNF concentration [34]. In a randomized, double-blind, placebo-controlled trial, 37 participants were asked to consume either freeze-dried blueberry beverage (24 g/day FDB, equivalent to one cup of fresh blueberries) or a blueberry placebo beverage for 90 days. Participants in the blueberry group showed significantly fewer repetition errors in a learning test and reduced switch cost in a task-switching test across study visits, relative to controls, but no improvement was observed in gait or balance. Moreover, in this study, no significant effect of blueberry consumption was observed on either the Geriatric Depression Scale or the Profile of Mood States [35].

The effect of blueberry consumption on cognitive function and mental health has been investigated in three studies. Whereas Miller et al. [35] reported no significant effect on the mood of the participants, Khalid et al. [7] reported a positive effect on the mood of both subject groups, referring to better positive affect scores. In contrast to the post consumption effect observed by Khalid et al., no long-term effect on mood was observed by Miller et al. after 45- and 90-days blueberry intervention. Bowtell et al. and Miller et al. also reported positive effects on cognitive function, referring to improvement in several tests.

## 4. Final Remarks

Strawberries (*Fragaria*) and Blueberries (*Vaccinium*) contain a wide variety of bioactive compounds, including many antioxidants with specific biochemical functions and beneficial health effects. The beneficial health effects are associated as protective effects against chronic diseases, such as cardiovascular disease, cancer, Alzheimer’s and other disorders. In this paper, nine studies investigating the effects of strawberry intervention and seven studies investigating the effect of blueberry intervention on inflammation, cardiovascular disease and cognitive function and mental health have been reviewed. The intervention type, the characteristics of participants, the format of berry and placebo consumption, the dose of berries and the main findings of the sixteen reviewed studies are summarized in Table 2 and Table 3.

Collectively, the sixteen investigated human intervention studies provide tendencies for an improvement in health by daily strawberry or blueberry consumption. In some studies, strawberry intervention selectively showed favorable effects on inflammation, insulin sensitivity, antioxidant status, insulin response and distribution or size of blood lipids, whereas some studies showed no effect on those attributes. Blueberry intervention showed positive effects in some cognitive tests and in some cases, selectively positive effects on blood pressure, endothelial function, insulin sensitivity or oxidative stress. However, several authors discussed the need for larger subject groups and longer trial periods to obtain more precise and consistent findings. Dose of anthocyanins, as well as age, overall health and predominantly sex of participants may have also influenced the results of the reviewed studies. It was also suggested to use fresh berries instead of powder. Most authors concluded that daily strawberry or blueberry consumption may have a positive effect on health., which should be investigated in further intervention studies.

## Figures and Tables

**Table 1:** Chemical composition of strawberry and blueberry [14].

	Strawberry	(Highbush) Blueberry
Flavonoids		
Anthocyanidins (mg/kg FW)	73.0	134
Flavanols (mg/kg FW)	9.1	1.1
Flavonols (mg/kg FW)	2.3	38.7
Phenolic acids		
Hydroxybenzoic acids (mg/kg FW)	5.7	1.5
Hydroxycinnamic acids (mg/kg FW)	7.1	135.0

**Table 2:** Effect of strawberry consumption on inflammation, cardiovascular disease and metabolic syndrome.

Intervention	Subjects	Format	Dose/day	Main findings	References
Post-prandial, randomized, single-blind, placebo-controlled, cross-over intervention	24 overweight males and females (BMI: 29.2 ± 2.3 kg/m^2^; age: 50.9 ± 15.0 year)	High-carbohydrate, moderate-fat meal + Berry group: Strawberry powder, water-based beverage Control group: Placebo drink	10 g powder	1. Attenuation of the postprandial inflammatory response 2. Reduction in postprandial insulin response 3. No differences for PAL-1, IL-1β and TNF-α	[17]
6-week, randomized, single-blind, placebo-controlled, parallel intervention + post-prandial high carbohydrates fat meal	24 overweight and obese males and females (BMI: 29.2 ± 2.3 kg/m^2^; age: 50.9 ± 15.0 year)	High-carbohydrate, moderate-fat meal + Berry group: Strawberry powder, water-based beverage Control group: Placebo powder, water-based beverage	10 g powder	1. Attenuation of the postprandial PAL-1 concentration and IL-1β response 2. No differences for platelet aggregation, hsCRP, TNF-α, insulin or glucose	[18]
Post-prandial, randomized, controlled, 4-arm, crossover intervention	21 males and females with abdominal obesity and insulin resistance (BMI 40.2 ± 7.2 kg/m^2^; age 39.8 ± 13.8 year)	High-fat breakfast + freeze-dried strawberry powder, beverage	Berry group: Group 1: 10 g Group 2: 20 g Group 3: 40 g Control group: 0 g powder (10 g powder is equivalent to 110 g fresh strawberries)	1. Reduction in postprandial plasma insulin concentrations, insulin: glucose ratio, and rate of glucose and insulin increase by 40 g FDS 2. Reduction in Oxidized low-density lipoprotein by 20 g FDS 3. IL-6 was not different among treatments	[19]
7-week, randomized, double-blind, placebo-controlled, cross-over intervention	20 obese males and females (13 Females: BMI, 35.6 ± 3.0 kg/m^2^; age, 31.8 ± 11.4 year; 7 Males: BMI, 32.3 ± 2.1 kg/m^2^; age, 29.4 ± 6.6 year)	Berry group: Strawberry powder, water-based beverage Control group: Placebo powder, water-based beverage	Powder equivalent to 320 g frozen strawberries	1. Reduction in serum cholesterol, small HDL particles, small HDL-cholesterol and Na and CO_2_ concentrations in the blood 2. Increase in mean particle size of LDL 3. No difference in blood pressure	[20]
12-week, randomized, controlled, parallel intervention	60 males and females with abdominal adiposity and elevated serum lipids LD-FDS: 15 subjects (BMI, 34.5 ± 4.4 kg/m^2^; age, 50 ± 10 year) HD-FDS: 15 subjects (BMI, 38.0 ± 7.1 kg/m^2^; age, 49 ± 11 year) LD-C: 15 subjects (BMI, 37.0 ± 4.4 kg/m^2^; age, 48 ± 10 year) HD-C: 15 subjects (BMI, 35.0 ± 5.2 kg/m^2^; age 48 ± 10 year)	Berry group: Freeze-dried strawberry powder, water-based beverage Control group: Placebo powder, calorie- and fiber-matched water-based beverage	Berry group: Group 1 (LD-FDS): 25 g (equivalent to 250 g fresh strawberries); Group 2 (HD-FDS): 50 g (equivalent to 500 g fresh strawberries)	1. Reduction in serum total and LDL-cholesterol, and NMR-derived small LDL particles by HD-FDS 2. Increase in Serum catalase activity by LD-FDS 3. Decrease in serum malondialdehyde by LD-FDS and HD-FDS 4. No effects on adiposity, blood pressure, glycemia, and serum concentrations of HDL cholesterol and triglycerides, C-reactive protein and adhesion molecules	[21]
6-week, randomized, double-blind, placebo-controlled, parallel intervention	36 males and females with type 2 diabetes (berry group: n = 19; BMI, 27.36 ± 4.23 kg/m^2^; age 51.9 ± 8.2 year; control group: n = 17; BMI, 28.58 ± 4.7 kg/m^2^; age 51.1 ± 13.8 year)	Berry group: Freeze-dried strawberry powder, water-based beverage Control group: Iso-caloric drink with strawberry flavoring	50 g powder	1. Increase of total serum antioxidant status 2. Decrease of serum malondialdehyde, glycated hemoglobin HbA1c, and hs-CRP concentrations 3. No effects on serum glucose concentrations and anthropometric indices	[22]
12-week, randomized, double-blind, placebo-controlled, cross-over intervention	17 males and females with radiographic evidence of knee OA (BMI: 39.1 ± 1.5 kg/m^2^; age: 57 ± 7 year)	Berry group: Freeze-dried strawberry powder, water-based beverage Control group: Placebo powder, water-based beverage	2 × 50 g powder	1. Decrease in interleukin (IL)-6, IL-1 and matrix metalloproteinase 2. Reduction in constant, intermittent, and total pain 3. No differences for hs-CRP, nitrite, glucose, and lipid profiles	[23]
12-week, randomized, controlled, parallel intervention	60 males and females with abdominal adiposity and elevated serum lipids (Age and BMI of subjects are identical to those in [21])	Berry group: Freeze-dried strawberry powder, water-based beverage Control group: Placebo powder, calorie- and fiber-matched water-based beverage	Berry group: Group 1 (LD-FDS): 25 g (equivalent to 250 g fresh strawberries); Group 2 (HD-FDS): 50 g (equivalent to 500 g fresh strawberries)	1. Increase in plasma antioxidant capacity 2. Increase in whole blood glutathione by LD-FDS and much higher by HD-FDS 3. No effects on glutathione peroxidase and glutathione reductase enzyme activities, plasma copper, iron, selenium and zinc	[27]
8-week, randomized, double-blind, placebo-controlled, parallel intervention	60 postmenopausal women (berry group 25 g FDS: n = 20; BMI, 31.0 ± 1.0 kg/m^2^; age 61 ± 1 year; 50 g FDS: n = 20; BMI, 32.7 ± 1.1 kg/m^2^; age 59 ± 1 year; control group: n = 20; BMI, 32.1 ± 0.7 kg/m^2^; age 58 ± 1 year)	Berry groups: Freeze-dried strawberry powder, water-based beverage Control group: Iso-caloric drink with strawberry flavoring	Berry group: Group 1: 2 × 25 g FDS (equivalent to three cups of sliced fresh strawberries); Group 2: 25 g FDS + 25 g placebo powder (equivalent to 1.5 cups of sliced fresh strawberries)	1. Decrease of systolic BP and brachial- and femoral-ankle pulse wave velocity by 25 g FDS 2. Increase of plasma nitric oxide metabolite levels by 50 g FDS 3. No effect on diastolic blood pressure and metabolites	[28]

**Table 3:** Effect of blueberry consumption on cardiovascular disease, metabolic syndrome, cognitive function, mood and associated risk factors.

Intervention	Subjects	Format	Dose/day	Main findings	References
6-week randomized, double-blind, placebo-controlled, parallel intervention	44 males and females with metabolic syndrome (Blueberry group: BMI 35.2 ± 0.8 kg/m^2^; age 55 ± 2 year; Control group: BMI 36.0 ± 1.1 kg/m^2^; age 59 ± 2 year)	Berry group: Blueberry powder, Smoothie Control group: Identical smoothie without blueberry bioactives	45 g powder	1. Improved reactive hyperemia index (endothiel function) 2. No difference in blood pressure and insulin sensitivity	[30]
6-week, randomized, placebo-controlled, crossover intervention	18 males with CVD risk factors (BMI 24.8 ± 2.6 kg/m^2^; age 47.8 ± 9.7 year)	Berry group: Wild blueberry (WB) powder, water-based beverage Control group: Placebo drink	25 g powder (equivalent to 148 g fresh wild blueberry)	1. Reduction of levels of endogenously oxidized DNA bases and levels of H2O2-induced DNA damage 2. No significant differences for markers of endothelial function and the other variables	[31]
1-week, randomized, single-blind, placebo-controlled, crossover intervention	19 females with at least two risk factors for type 2 diabetes (BMI: 31.4 ± 2.9 kg/m^2^, age: 53 ± 6.3 year)	Berry group: Wild blueberry juice Control group: Placebo beverage	240 mL juice	1. Trend for lowering systolic blood pressure and increased serum concentrations of nitrates and nitrites 2. No changes in glucose, insulin, insulin sensitivity, triglycerides, inflammatory markers, adhesion molecules, oxidative stress, endothelial function or blood pressure	[32]
8-week, randomized, double-blind, placebo-controlled, parallel intervention	48 postmenopausal women with pre- and stage 1-hypertension (Blueberry group: BMI 30.1 ± 5.94 kg/m^2^; age 59.7 ± 4.58 year; Control group: BMI 32.7 ± 6.79 kg/m^2^; age 57.3 ± 4.76 year)	Berry group: Freeze-dried blueberry powder, water-based beverage Control group: Placebo drink	22 g powder (equivalent to one fresh cup blueberries)	1. Decrease in systolic blood pressure, diastolic blood pressure and brachial-ankle pulse wave velocity 2. Increase in Nitric oxide levels	[33]
1: Randomized, double-blind, placebo-controlled, crossover intervention 2: Randomized, double-blind, placebo-controlled intervention	1:21 males and females (age: 20.1 ± 1.0) 2:52 males and females (age: 8.2 ± 1.0)	Berry group: Freeze-dried WB powder + low-flavonoid Rocks Orange Squash, water-based beverage Control group: Vitamin C and sugar matched placebo drink + 30 mL low-flavonoid Rocks Orange Squash, water-based beverage	30 g powder 30 mL Orange Squash	1. Increase in post-consumption positive affect score 2. No effect on negative affect score 3. Session reduction of NA after both blueberry and placebo consumption	[7]
12-week, randomized, double-blind, placebo-controlled intervention	26 males and females(Blueberry group: BMI 25.9 ± 3.3 kg/m^2^, age 67.5 ± 3.0 year; Control group: BMI 27.1 ± 4.0 kg/m^2^, age 69.0 ± 3.3 year)	Berry group: Blueberry concentrate Control group: Placebo concentrate	30 mL concentrate (providing 387 mg anthocyanidins)	1. Increase in brain activation responses and resting-state perfusion in the gray matter of the parietal and occipital lobes 2. Decrease in serum glutathione concentration 3. No differences in brain activity, resting state perfusion in the frontal lobes, protein carbonylation HNE adduct or malonaldehyde formation and serum hsCRP or BDNF concentration	[34]
90 days, randomized, double-blind, placebo-controlled intervention	37 males and females with English fluency, ability to walk 20 min unassisted, and > 12 months postmenopausal (Blueberry group: BMI 24.1 ± 3.7, age 67.8 ± 4.6 year, Placebo group: BMI 24.0 ± 2.5, age 67.3 ± 4.8 year)	Berry group: Freeze-dried blueberry powder Control group: Placebo powder	24 g powder (equivalent to one cup of fresh blueberries)	1. Decrease in repetition errors and reduction in switch cost 2. No improvement in gait or balance 3. No effect of Geriatric Depression Scale or the Profile of Mood States	[35]
